# Clinicopathologic characteristics and outcomes of lupus nephritis with positive antineutrophil cytoplasmic antibody

**DOI:** 10.1080/0886022X.2020.1735416

**Published:** 2020-03-04

**Authors:** Shuai Wang, Jin Shang, Jing Xiao, Zhanzheng Zhao

**Affiliations:** Department of Nephrology, The First Affiliated Hospital of Zhengzhou University, Zhengzhou, China

**Keywords:** Lupus nephritis, anti-neutrophil cytoplasmic antibody, clinicopathologic characteristics, pulmonary infection, prognosis

## Abstract

**Aims:**

The aim was to determine whether anti-neutrophil cytoplasmic antibody (ANCA)-positive serology in patients with lupus nephritis (LN) is associated with different clinicopathologic features and outcomes.

**Methods:**

In our retrospective analysis, 283 patients were enrolled between 2013 and 2018. Thirty-six patients were ANCA-positive, and this group was compared with the remaining 247 patients who were confirmed as ANCA-negative at the time of biopsy.

**Results:**

ANCA-positive LN patients exhibited higher anti-dsDNA antibody titers and serum creatinine levels and lower serum hemoglobin concentrations than ANCA-negative LN patients. On pathological evaluation, segmental endocapillary hypercellularity observed by light microscopy was significantly more common in the ANCA-positive group. This feature was not significantly different in the treatment group, but the response to treatment was significantly different, as was remission (76.1% vs 69.4%, *p* < 0.001), between the ANCA-negative and ANCA-positive groups. During follow-up, the times to renal replacement therapy (RRT) and death were significantly different between the two unmatched groups (chi-square test, *p* = 0.041). Multivariate Cox analysis revealed that neurological disorders, ANCA positivity, and the chronicity index (CI) remained independent risk factors for patient survival. Pulmonary infection was the main cause of death and was most often due to fungal infection.

**Conclusion:**

ANCA-positive LN patients typically exhibited higher anti-dsDNA antibody titers, lower serum hemoglobin concentrations and worse renal function than ANCA-negative LN patients. Fungal infection was the main cause of death. We observed that ANCA positivity was an independent risk factor for patient survival.

## Introduction

Lupus nephritis (LN) is a major complication of systemic lupus erythematosus (SLE) that occurs in up to two-thirds of SLE patients, and the overall 5-year survival rate is 94%. Some studies reported that class IV nephritis, elevated anti-DNA antibody titers, and hypocomplementemia were risk factors for renal failure and death [1].

Notably, anti-neutrophil cytoplasmic antibodies (ANCAs) are detected in approximately 16–42% [2,3] of patients with SLE and in 37–53% [4,5] of patients with LN. ANCA-associated glomerulonephritis (GN) is characterized by the presence of ANCAs against myeloperoxidase (MPO) or proteinase 3 (PR3) in the serum of patients with GN [6]. Subclinical MPO-ANCA levels could distinguish SLE without LN in the future [7]. The extensive necrotic and pauci-immune glomerular inflammation observed in some patients with Class IV-S LN may be associated with ANCAs [5,6,8–10]. Some studies have reported an association between this histopathologic phenotype and ANCA positivity [5,6,8,9,11–13], but others found no association.

Treatment for LN and ANCA-associated vasculitis is still dominated by immunosuppressive therapy to improve short-term survival, but the adverse effects of infection, which is the main risk of death, affect long-term survival [14].

The aim of our study was to establish whether a relationship exists between ANCAs and the histopathologic features of LN, serologic SLE activity, and renal outcomes in a Chinese population.

## Methods

### Patients

This study was a retrospective review of our cohort of patients with LN who were identified in our hospital renal biopsy database at the First Affiliated Hospital of Zhengzhou University from 14 December 2012 to 23 July 2018. The included patients fulfilled the following criteria [1]: met the ISN/RPS 2003 diagnostic criteria [2,15] were tested for ANCAs by both an indirect immunofluorescence assay and an enzyme-linked immunosorbent assay (ELISA) or flow immunofluorescence [3], had renal involvement and a renal biopsy specimen containing ≥10 glomeruli under light microscopy, and [4] were older than 18 years but younger than 60 years. Patients with any of the following conditions were excluded [1]: drug-associated [16] (hydralazine, propylthiouracil, minocycline, and levamisole-adulterated cocaine) LN, cancer, cryoglobulinemia, or infection [2]; comorbid kidney diseases, such as IgA nephropathy, diabetic nephropathy, membranous nephropathy, and anti-glomerular basement membrane nephritis; and [3] hepatitis B virus, hepatitis C virus, or HIV infection.

This study was approved by the Institutional Review Board of the First Affiliated Hospital of Zhengzhou University (2019-KY-134), and informed consent was not required (version number: V1.0, version date: 20 March 2019). Positivity for ANCAs was based on an elevated titer for anti-MPO or anti-PR3 antibodies, as detected by ELISA or flow immunofluorescence (Zeus Scientific, Inc; Branchburg, NJ, USA; using the normal range determined by our laboratory) [17].

### Renal histopathology

Renal biopsy specimens were examined by light microscopy, immunofluorescence, and electron microscopy according to standard procedures. Renal biopsy reports were reviewed to identify the following [1]: the class of LN, on the basis of the 2003ISN/RPS classification system [2]; the presence of necrosis [3]; the presence of crescents, including their classification, defined as cellular, small cellular, fibrous, small fibrous, cellular fibrous, or small cellular fibrous [4]; the pattern of endocapillary hypercellularity on light microscopy, which was defined as segmental, global, or absent [5]; the extent of subendothelial electron-dense deposits on electron microscopy (EM), defined as present or absent and serving as a marker of immune complex deposition in glomerular capillaries [6]; extracapillary proliferation, including mesangial cell proliferation and mesangial matrix proliferation, which was defined as mild, diffuse or other, including focal segmental mild, focal segmental mild to moderate, mild to moderate and moderate to severe [7]; the presence of sclerosis; and [8] the activity index (AI) and chronicity index (CI) of renal biopsy specimens in LN.

All renal biopsies were read independently by two experienced renal pathologists who were blinded to the clinical data. Inconsistencies in renal reports were resolved by discussion and consensus.

### Clinical and laboratory parameters

The patients’ clinical data were collected retrospectively at the time of biopsy and included extrarenal manifestations (fever, thrombocytopenia, leukocytopenia, pleuritis, mouth ulcers, malar rash, alopecia, myositis, arthritis, vasculitis, neurological disorders, and photosensitivity) and blood and urine test results (double-stranded deoxyribonucleic acid (dsDNA), MPO quantitative and qualitative results, PR3 quantitative and qualitative results, serum C4, serum C3, C-reactive protein (CRP), erythrocyte sedimentation rate (ESR), serum creatinine (CREA), blood urea nitrogen, uric acid (UA), serum albumin, blood glucose, serum cholesterol, triglycerides (TG), white blood cell (WBC) counts, red blood cell (RBC) counts, hemoglobin (Hb), platelets (PLT), urine erythrocyte counts, urine leukocyte counts, urine cast, and 24 h total protein (24 h TP). Both the treatments in use at the time of biopsy and those given in response to the biopsy in question were also reviewed. The treatments in use were standardized in our department depending on the clinical scenario and the clinicians’ judgment, as follows: steroids only, steroids combined with cyclophosphamide, steroids combined with mycophenolate mofetil, and other treatments, including steroids combined with tacrolimus, steroids combined with mycophenolate mofetil and tacrolimus, dialysis and plasma exchange. Remission included complete remission and partial remission. Complete remission was defined as a 24-h TP level of less than 0.3 g and a normal serum albumin level. Partial remission was defined as a decrease in urinary protein excretion by >50% to <3 g per 24 h, with a serum albumin of ≥30 g/L and a normal serum creatine level or an elevation of <10% above the baseline value [1,11,15]. Survival data were collected retrospectively to determine patient survival independent of renal replacement therapy (RRT) between the time of biopsy and 31 March 2019, when all data were censored. The median follow-up times for patients with ANCA-positive biopsy specimens (ANCA-positive group) and ANCA-negative biopsy specimens (ANCA-negative group) were 45.951 months and 71.158 months, respectively. The patients’ demographic and clinical records included age, sex, and ethnicity.

### Statistical analyses

All data were analyzed using IBM SPSS 20.0. Quantitative data are expressed as the means ± SDs and medians (interquartile ranges). Categorical variables are expressed as frequencies. The 1-sample Kolmogorov-Smirnov test was used to assess the normality of distribution. According to the normality of their distributions, continuous variables were compared with the independent sample *t*-test. The Kruskal–Wallis H test was used to compare nonnormally distributed continuous data. Categorical data were compared using the chi-squared test. Kaplan–Meier survival analysis was used to estimate patient and renal survival, and the log-rank test was used to analyze the differences between the survival curves. A multivariate Cox regression model was used to evaluate the risk factors for poor outcomes. Relevant variables that were significantly associated with poor outcomes in univariate analysis were included in multivariate models. The final result was adjusted for neurological disorder and the CI. The results are expressed as relative ratios (RR) with 95% confidence intervals.

All analyses were performed comparing the ANCA-positive group with the ANCA-negative group unless Cox regression was performed. All tests were 2-sided, and *p* < 0.05 was considered significant.

## Results

In our retrospective analysis, 283 patients were enrolled between 2013 and 2018. Thirty-six patients were ANCA-positive, and this group was compared with the remaining 247 patients who were confirmed as ANCA-negative at the time of biopsy (Table 1). The majority of patients in the ANCA-positive group had anti-myeloperoxidase (MPO) antibodies (82%) at the time of biopsy, 11% had anti-proteinase-3 (PR3) antibodies, and 7% had both anti-MPO and anti-PR3 antibodies (Table 1). Patients in the ANCA-positive group had significantly higher MPO and PR3 titers at the time of biopsy than patients in the ANCA-negative group by flow immunofluorescence (median [range]: 144.0 U/mL [66.9–206.5] vs. 34.0 U/mL [16.0–54.0], *p* < 0.001) and (median [range]: 23.5 U/mL [12.3–42.9] vs. 11.0 U/mL [7.0–27.0], *p* = 0.033). MPO and PR3 titers were not significantly different between the ANCA-positive and ANCA-negative groups at the time of biopsy, as indicated by ELISA (Table 1).

**Table 1.** Patient demographic characteristics at the time of renal biopsy.

Demographic data	ANCA-positive group (n = 36)	ANCA-negative group (n = 247)
Median age (range)	32 (18–57)	32 (18–58)
At biopsy, years		
Sex (M:F)	1:4.6	1:35
MPO titers, U/mL (0–20)	5.5 (3.9–7.4)	11.2 (5.7–229.4)
PR3 titers, U/mL (0–20)	6.1 (4.2–7.8)	7.5 (5.4–31.4)
MPO titers, U/mL (0–100)	144.0 (66.9–206.5)	34.0 (16.0–54.0)
PR3 titers, U/mL (0–100)	23.5 (12.3–42.9)	11.0 (7.0–27.0)
ANCA serology, %	MPO: 82	
	PR3: 11	
	MPO and PR3: 7	

ANCA: antineutrophil cytoplasmic antibody; F: female; M: male; MPO: myeloperoxidase; PR3: proteinase 3. MPO and PR3 reference value range: 0–20 U/mL (ELISA) or 0–100 U/mL (flow immunofluorescence).

### Demographic characteristics of the patient population

Age at the time of biopsy was not significantly different between the ANCA-positive and ANCA-negative groups (*p* = 0.749). The male:female ratio of the ANCA-positive group was significantly higher than that of the ANCA-negative group (1:4.6 vs 1:35, *p* = 0.021; Table 1). The affected individuals were predominantly young women, and all patients were Chinese.

### Histopathologic features

**Figure 1.**
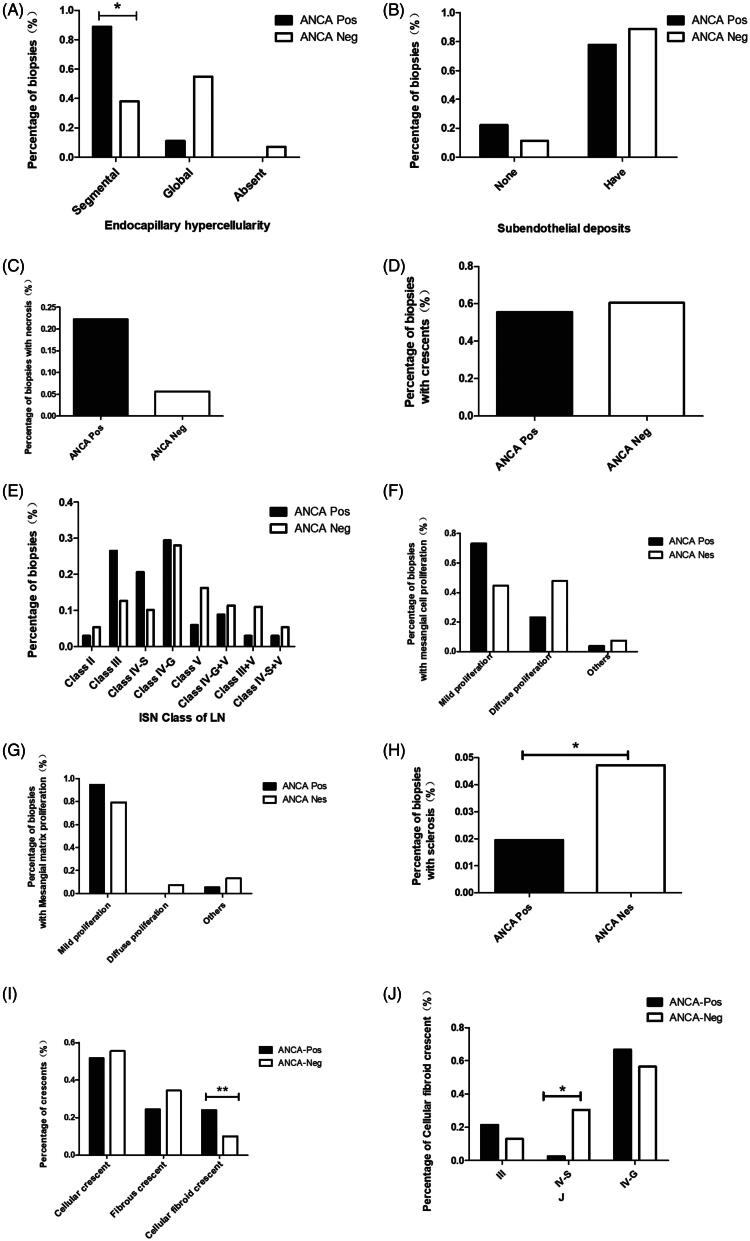
Histopathological features of proliferative LN.

Comparing the biopsy specimens with proliferative LN (Class III and Class IV) between the 2 groups, segmental endocapillary hypercellularity on light microscopy was significantly more common in the ANCA-positive group than in the ANCA-negative group (88.9% vs. 38.0%, *p* = 0.011; Figure 1(A)). There was no significant difference in the extent of subendothelial electron-dense deposits between the 2 groups, as determined by EM (*p* = 0.688; Figure 1(B)). The proportions of biopsy specimens with necrosis (5.6% vs. 22.2%, *p* = 0.268; Figure 1(C)) and crescent formation (60.1% vs. 55.6%, *p* = 1; Figure 1(D)), as well as the stage (Figure 1(E)), mesangial cell proliferation (*p* = 0.051; Figure 1(F)) and mesangial matrix proliferation (*p* = 0.219; Figure 1(G)) were comparable between the ANCA-negative and ANCA-positive groups. Therefore, these differences did not reach statistical significance. The percentage of glomerulosclerosis at the time of biopsy was significantly lower in the ANCA-positive group than in the ANCA-negative group (*p* = 0.046; Figure 1(H)).

There were significant differences between the ANCA-positive and ANCA-negative groups in the types of crescents (*p* < 0.001; Figure 1(I)) and in the presence of cellular fibroid crescents and cellular and fibrous crescents (*p* < 0.001; Figure 1(I)); the IV-S group had a significantly lower proportion of cellular fibroid crescents than the other groups (*p* = 0.016; Figure 1(J)).

### Serological features

**Figure 2.**
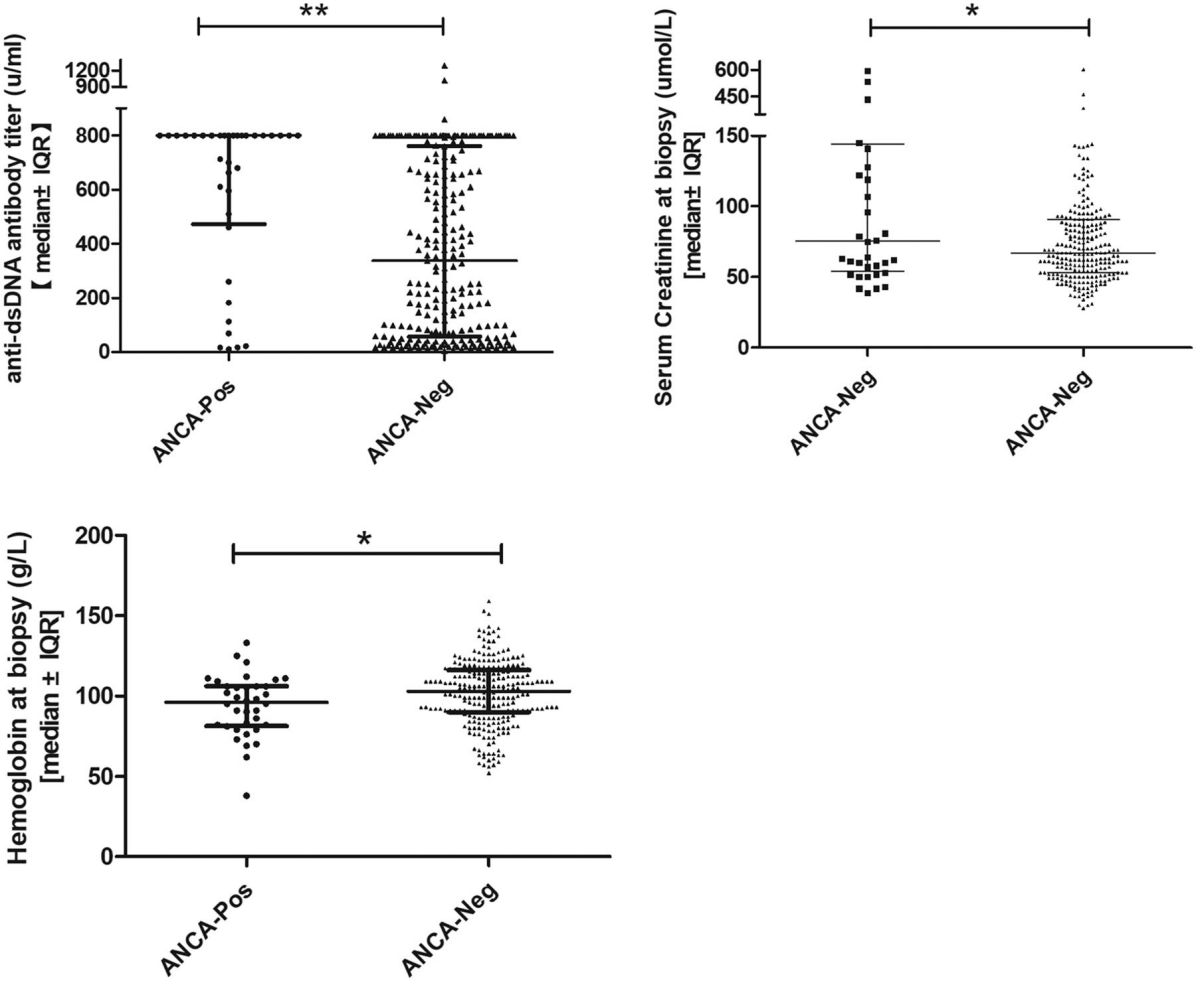
Serological features at the time of biopsy.

Anti-dsDNA antibody titers, serum complement, and serum hemoglobin concentrations were used as serologic markers of SLE activity and were compared between the 2 groups at the time of biopsy. Patients in the ANCA-positive group had significantly higher anti-dsDNA antibody titers at the time of biopsy than those in the ANCA-negative group (median [range]: 800.0 units/ml [10–800] vs. 337.2 u/ml [4.6–1296.2], *p* < 0.001; Figure 2(A); Table 2). Similarly, the serum hemoglobin concentration was significantly lower at the time of biopsy in the ANCA-positive group than in the ANCA-negative group (median [range]: 96 g/l [38–133] vs. 103 g/l [52–159], *p* = 0.033; Figure 2(C); Table 2). There was no significant difference between the ANCA-positive and ANCA-negative groups in terms of the serum C4 and C3 concentrations at the time of biopsy (median [range]: 0.105 g/l [0.01–0.28] vs. 0.08 g/l [0.01–0.46], *p* = 0.668; median [range]: 0.54 g/l [0.15–1.86] vs. 0.48 g/l [0.11–1.67], *p* = 0.189; Table 2).

**Table 2:** Patient parameters at the time of renal biopsy.

Parameters	ANCA-Positive (N = 247)	ANCA-Negative (N = 36)	*p* Value
Systolic pressure, mmHg	125 (114, 137)	129 (119, 143)	0.163
Diastolic pressure, mmHg	81 (74, 92)	85 (76, 94)	0.594
Average hospital stay, days	10.00 (6.13, 17.50)	8.50 (6.00, 13.00)	0.102
SLEDAI	8 (6, 12)	8 (5, 12)	0.944
ds-DNA, u/mL	800.00 (472.33, 800.00)	337.20 (58.00, 760.20)	<0.001
C4, g/L	0.11 (0.06, 0.16)	0.08 (0.04, 0.17)	0.668
C3, g/L	0.54 (0.33, 0.82)	0.48 (0.31, 0.70)	0.189
CRP, mg/L	3.06 (1.04, 10.65)	1.94 (1.00, 5.20)	0.385
ESR, mm/h	52.50 (24.25, 84.50)	46.00 (21.00, 85.00)	0.330
CREA, µmol/L	75.50 (54.00, 144.00)	67.00 (53.00, 91.00)	0.050
Urea, mmol/L	8.25 (5.13, 12.15)	6.20 (4.30, 9.50)	0.974
UA, µmol/L	379.50 (277.25, 416.00)	339.00 (267.00, 435.00)	0.451
ALB, g/L	27.95 (22.45, 31.75)	25.00 (20.50, 31.10)	0.242
Glu, mmol/L	4.57 (4.27, 4.86)	4.50 (4.10, 4.90)	0.974
T-CHO, mmol/L	4.85 (3.55, 6.23)	5.10 (4.00, 6.60)	0.500
TG, mmol/L	2.11 (1.28, 2.54)	1.90 (1.34, 2.90)	0.654
WBC, 10^9^/L	4.90 (3.15, 8.88)	4.60 (3.40, 6.40)	0.175
RBC, 10^12^/L	3.27 ± 0.67	3.44 ± 0.68	0.148
Hb, g/L	93.83 ± 19.02	101.54 ± 20.18	0.033
PLT, 10^9^/L	150.00 (104.25, 206.50)	155.00 (108.00, 208.00)	0.960
24h TP, g	2.16 (1.22, 3.88)	3.11 (1.54, 5.78)	0.145

SLEDAI: SLE disease activity index; ds-DNA: double-stranded deoxyribonucleic acid; C4: serum C4; C3: serum C3; CRP: C-reactive protein; ESR: erythrocyte sedimentation rate; CREA: serum creatinine; Urea: blood urea nitrogen; UA: uric acid; ALB: serum albumin; Glu: blood glucose; T-CHO: serum cholesterol; TG: triglyceride; WBC: white blood cell; RBC: red blood cell; Hb: hemoglobin; PLT: platelet; 24 h TP: 24 h total protein.

### Renal function and proteinuria

Patients in the ANCA-positive group had significantly worse renal impairment at the time of biopsy than those in the ANCA-negative group (median serum creatinine level, 75.5 µmol/l vs. 67.0 µmol/l, *p* = 0.055; Table 2). However, both groups contained patients with advanced renal impairment as well as patients with relatively normal renal function (range: serum CREA 28–604 µmol/l in the ANCA-positive group and 39–595 µmol/l in the ANCA-negative group; Figure 2(B)).

Patients in the 2 groups had no significant proteinuria at the time of biopsy (24 h protein, median [range]: 2.16 g/24 h [0.13–21.05] in the ANCA-positive group vs. 3.11 g/24 h [0.02–17.52] in the ANCA-negative group; *p* = 0.145; Table 2).

### Treatment and extrarenal manifestations

The treatments were not significantly different between the two groups (*p* = 0.552; Table 3), and the same was true for extrarenal manifestations (Table 4). However, the response to treatment was significantly different between the two groups (*p* = 0.012; Table 3). Patients in the ANCA-positive group had significantly higher remission rates than those in the ANCA-negative group (76.1% vs 69.4, *p* < 0.001; Table 3).

**Table 3.** Treatment at the time of renal biopsy and follow-up data.

	ANCA-Positive	ANCA-Negative	*p* Value
	(n = 36)	(n = 247)	0.552
Steroid only	20 (55.6%)	132 (53.4%)	
Steroid + cyclophosphamide	5 (13.9%)	40 (16.2%)	
Steroid + mycophenolate mofetil	9 (25.0%)	44 (17.8%)	
Others	2 (5.5%)	31 (12.6%)	
Response			0.012
Remission	25 (69.4%)	185 (74.9%)	
Death	5 (13.9%)	5 (2.0%)	
ESRD	2 (5.6%)	6 (2.4%)	

Others include Steroid + tacrolimus, Steroid + mycophenolate mofetil + tacrolimus, dialysis and plasma exchange.

**Table 4.** Patient extrarenal manifestations at the time of renal biopsy.

	ANCA-Positive (n = 36)	ANCA-Negative (n = 247)	*p* Value
Fever	7	31	0.383
Thrombocytopenia	7	54	0.742
Leukocytopenia	8	33	0.158
Pleuritis	2	5	0.219
Mouth ulcer	1	8	1
Malar rash	5	42	0.639
Alopecia	2	13	1
Myositis	1	1	0.239
Arthritis	5	19	0.354
Vasculitis	0	2	1
Neurological disorder	1	2	0.336
Photosensitivity	1	4	0.496

### Long-term outcomes

The recorded events were patient death and the start of RRT, which was either at dialysis or transplantation, with a dialysis time longer than 3 months [1]. If patients were lost to follow-up or an event did not happen by 31 March 2019, when the outcomes for all patients were analyzed, the data were censored. There were 7 events in the ANCA-positive group and 11 events in the ANCA-negative group over the entire duration of follow-up. Of the 7 patients who experienced events in the ANCA-positive group, 5 died due to pulmonary infection, and 2 remained on RRT. Of the 11 patients who experienced events in the ANCA-negative group, 5 died; one died from pulmonary infection, two died from lupus encephalopathy, and two died from other causes. The other 6 patients remained on RRT. The rate of mortality due to pulmonary infection was significantly higher in the ANCA-positive group than in the ANCA-negative group (chi-square test, *p* = 0.013).

Regarding the cases of respiratory failure and eventual death caused by pulmonary infection, the relevant characteristics are shown in Table 5. The prominent features were mainly MPO-associated LN, and the pathological types were mainly proliferative lupus, especially type IV-G. The survival time was short, with a median survival time of 2.5 months. All of the deaths were attributed to respiratory failure caused by pulmonary infection, and fungal infection accounted for a large proportion of these cases.

**Table 5.** Clinicopathologic feature of death owing to pulmonary infection.

	1	2	3	4	5	6
Sex	Male	Female	Female	Female	Female	Female
Age(year)	25	31	24	47	37	44
ANCA	Negative	MPO-Pos	MPO-Pos	MPO-Pos	PR3-Pos	MPO-Pos
ds-DNA (u/mL)	357.7	800	17.2	800	800	800
Hb (g/L)	107	106	67	97	90	92
CREA, µmol/L	456	60	534	202	100	135
Pathological type	IV-G(A/C)	III-(A)	IV-G(A/C)	IV-G(A/C)	IV-G(A)+V	IV-G(A)
AI	12	6	6	15	13	16
CI	6	1	5	4	1	0
Treatment plan	Full dose prednisone	Full dose Methyl prednisolone	Full dose prednisone + CTX 0.8 g ivgtt	0.25 g × 3 days Full dose prednisone + MMF 0.5 g bid	0.25 g × 8 days CTX 0.4 g ivgtt Full dose Methyl prednisolone	0.25 g × 3 days Full dose Methyl prednisolone
Follow-up (months)	5	5	2	0.5	3	2
Pulmonary disease	Fungal infection	Bacterial infections	Fungal infection	Bacterial infections	Fungal infection	Fungal infection

The first line of numbers represents the identity of each patient; Pos: Positive.

**Figure 3.**
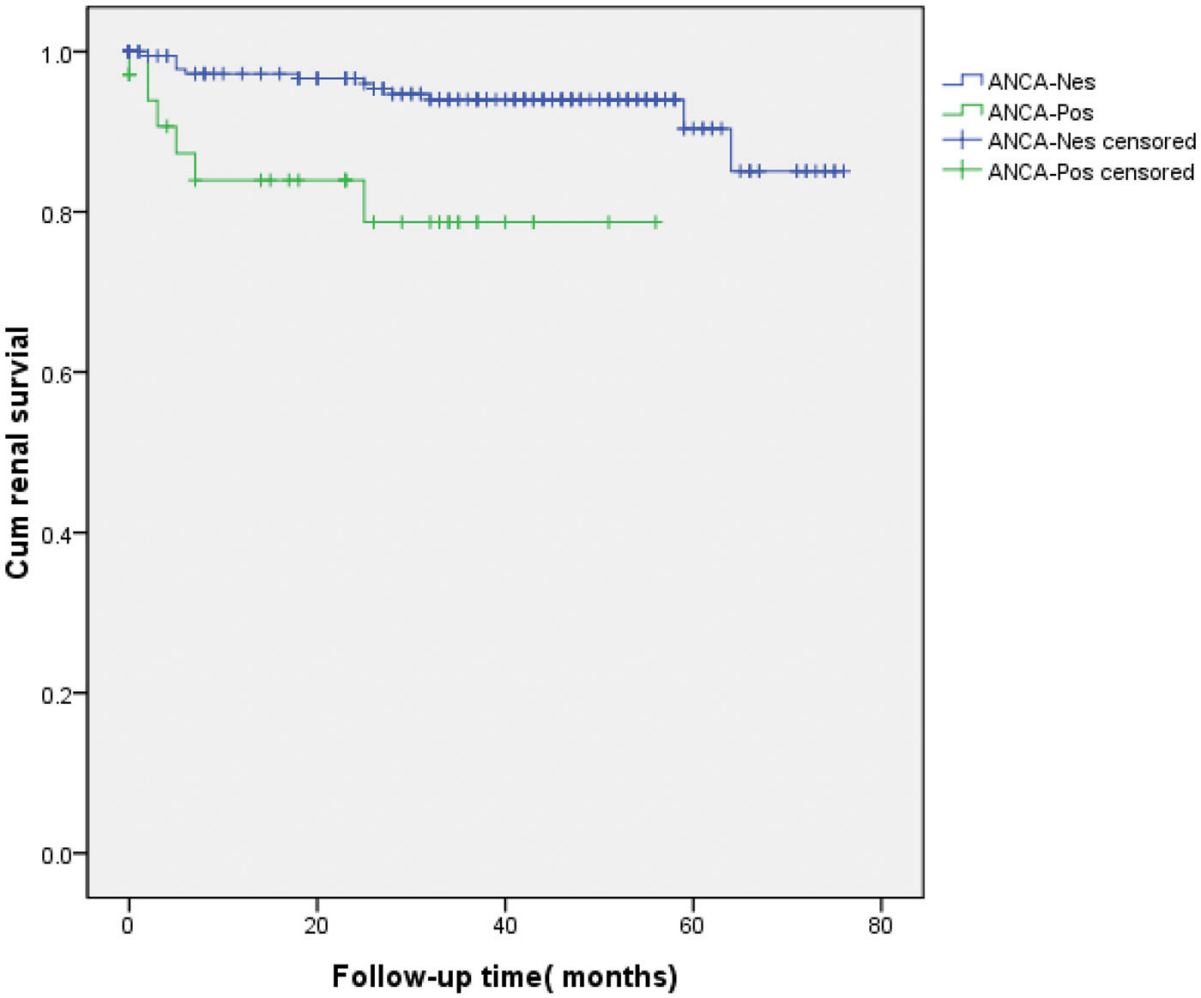
Kaplan–Meier survival curve illustrating patient survival independent of renal replacement therapy (RRT; all patients shown).

The time to RRT or death was significantly different between the two unmatched groups (chi-square test, *p* = 0.041; Figure 3), as determined by Kaplan–Meier analysis (log-rank test, *p* = 0.002; Figure 3). Univariate Cox regression analysis showed that treatment, systolic pressure, diastolic pressure, neurological disorders, WBC counts, RBC counts, Hb, PLT, ANCAs, dsDNA, C3, C4, ESR, CRP, Glu, urea, CREA, UA, TG, T-CHO, 24 h TP, class of LN, CI, endocapillary hypercellularity, and crescent formation were risk factors for LN patient survival. Multivariate Cox analysis revealed that neurological disorders (RR, 6.443; 95% confidence interval, 1.447–28.592; *p* = 0.014), ANCAs (RR, 3.582; 95% confidence interval, 1.273–10.076; *p* = 0.016), and the CI (RR, 1.538; 95% confidence interval, 1.294–1.828; *p* = 0.014) remained independent risk factors for patient survival after adjusting for WBC counts, dsDNA, ESR, and CREA (Table 6).

**Table 6.** Univariate and multivariate Cox regression analysis of poor renal outcomes.

Characteristics	Univariate	Multivariate	Corrected result	
	*p* Value	*p* Value	*p* Value	RR (95% CI)
Treatment	0.083			
Average hospital stay, days	<0.001			
Systolic pressure, mmHg	<0.001			
Diastolic pressure, mmHg	0.015			
Neurological disorder	<0.001	0.004	0.014	6.443 (1.447–28.592)
WBC, 10^9^/L	0.073	0.006		
RBC, 10^12^/L	<0.001			
Hb, g/L	<0.001			
PLT, 10^9^/L	<0.001			
ANCA	0.001	0.013	0.016	3.582 (1.273–10.076)
ds-DNA, U/mL	<0.001	0.014		
C3, g/L	0.001			
C4, g/L	<0.001			
ESR, mm/h	<0.001	0.018		
CRP, mg/L	<0.001			
Glu, mmol/L	<0.001			
Urea, mmol/L	<0.001			
CREA, µmol/L	<0.001	0.014		
UA, µmol/L	<0.001			
TG, mmol/L	<0.001			
T-CHO, mmol/L	<0.001			
24h TP, g	<0.001			
Class of LN	0.085			
AI	0.017			
CI	<0.001	0.014	<0.001	1.538 (1.294–1.828)
Endocapillary hypercellularity	0.073			
Crescent formation	0.021			

## Discussion

In our study, the positive rate for ANCA was 12.7% (36 of 283 patients). The predominant ANCA pattern observed was p-ANCA, of which 89% of cases had anti-MPO antibodies. Based on our findings, flow immunofluorescence is recommended for ANCA detection. Regarding clinical features, ANCA-positive LN patients had more active lupus and worse renal function. Fungal infection was the main cause of death. Moreover, ANCA-positive LN patients showed a rapidly progressive or chronic injury. We observed that ANCA was an independent risk factor for patient survival.

Our results suggest that patients with LN and positive ANCA serology are more likely than ANCA-negative patients to have segmental endocapillary hypercellularity and cellular fibroid crescents on renal biopsy (ISN/RPS Classes III, IV-S, IV-G LN), a finding that has also been reported by others [1,11,18]. In our study, there was no significant difference in extracapillary proliferation between the ANCA-positive and ANCA-negative groups. Patients with LN and positive ANCA serology had less diffuse extracapillary proliferation than ANCA-negative patients. Similarly, the percentage of glomerulosclerosis was significantly lower in the ANCA-positive group at the time of biopsy than in the ANCA-negative group. However, Yuan Wang et al. reported higher glomerulosclerosis rates in the ANCA-positive group [1,11]. The reason may be the inclusion of different pathologies or regions in the analysis. The proportion of glomerular necrosis was higher in the ANCA-positive group than in the negative group; however, this difference was not statistically significant. Cui Li et al. reported that the higher rate of glomerular necrosis was statistically significant. There are also reports that the CI, the AI, tubular atrophy, and interstitial fibrosis were significantly different between the two groups [1]. In summary, patients with LN and positive ANCA serology have rapidly progressive or chronic injury. Due to the uncertainty of pathological characteristics, this result suggests that patients with LN accompanied by ANCA positivity should undergo renal biopsy as soon as possible under the permitted conditions to clarify the pathological type and decide the treatment plan.

In this study, we found that compared with negative ANCA serology, positive ANCA serology at the time of biopsy appeared to be associated with serologically more active lupus (higher dsDNA titers and lower serum Hb concentrations) and worse baseline renal function, a finding that has also been reported by others [19]. This study suggests that we should pay more attention to these patients and strengthen management to help patients understand the characteristics of this disease and cooperate with treatments. Others reported additional results, such as lower C3 and C4 and higher urinary RBC counts in patients with positive ANCA serology [1,11,18]. However, our results showed that complement levels were higher in the ANCA-positive group than in the ANCA-negative group but did not reach statistical significance. This result may be due to demographic characteristics, the application of different classifications, and the patient selection criteria.

The treatment and extrarenal manifestations [1] at the time of renal biopsy were not significantly different between the ANCA-positive and ANCA-negative groups. Some studies reported that photosensitivity, oral ulcerations, and alopecia were more common in the ANCA-positive group than in the ANCA-negative group [11], and others observed a higher remission rate and better prognoses when using mycophenolate mofetil (MMF) than when using cyclophosphamide as induction therapy for ANCA-positive LN patients. It was also reported that tacrolimus might be a useful immunosuppressant for patients with progressive LN and MPO-ANCAs [13,20]. The above results suggest that a preference for tacrolimus or MMF is indicated during drug selection for ANCA-positive patients.

LN and ANCA positivity were responsible for the severe clinical course [10,21,22]. Neurological disorders, ANCAs, and the CI remained independent risk factors for patient survival in this study. The neutrophil-to-lymphocyte ratio, ANCAs and the estimated glomerular filtration rate (EGFR) were reported to be independent risk factors for patient survival in another article [11]. Other studies also reported poor prognoses with increased ANCA values [13,18]. Among the results of the above studies, the CI is an index of renal biopsy, demonstrating the necessity of renal biopsy in LN. It is also suggested that the ANCA value should be dynamically monitored in clinical work to predict prognosis in LN. Our findings indicate that further prospective studies are needed to assess the effects of positive ANCA serology on outcomes.

Death due to pulmonary infection was significantly more common in the ANCA-positive group than in the ANCA-negative group [23]. There was a high probability of the pathogenic organism being a fungus (66.7%). For one patient with LN and MPO seropositivity, methylprednisolone pulse therapy and intravenous cyclophosphamide followed by oral prednisone was initiated, followed by intermittent hemodialysis; the patient ultimately died due to Candida pneumonia. Another patient with the same diagnosis who was treated with plasma exchange and methylprednisolone pulse therapy died of recurrent pulmonary hemorrhage and concurrent cryptococcal pneumonia [10,22]. This result may be explained by the occurrence of immunosuppression-related adverse events such as neutropenia and infection. It is suggested that when using glucocorticoids, immunosuppressive drugs, and cytotoxic drugs, pulmonary condition should be monitored. Furthermore, patients should be informed that if respiratory tract infections occur, they should stop these drugs and report to the hospital in a timely manner to prevent the occurrence of dangerous events. Therefore, targeted therapy may be an option.

LN processes are characterized by neutrophils and C3 deposits in the glomeruli and systemic C3 depletion. In MRL/lpr mice, inhibition of C5aR activation can reduce glomerular inflammation and kidney disease and prolong survival [24]. A human trial with eculizumab (anti-C5) has shown preliminary efficacy [25].

ANCAs are characterized by a paucity of immunoglobulin deposits [26]. Murine models demonstrated activation of the alternative complement pathway, and C5 is essential for disease induction, with C5a as a key mediator [27,28]. Blockade of C5aR1 prevented disease expression, and C5aR2 deficiency aggravated the disease condition; antagonism is being tested as a therapy for patients with ANCA-associated vasculitis [26,29].

C5aR is a target of both ANCA and LN and thus has research prospects. The spleen tyrosine kinase (SYK) inhibitors RTX, TKI, and IL-17C/IL-17RE may inform the development of future clinical studies in this field [30–33], and targeted therapy will become mainstream in the future.

This report has some limitations, mainly associated with its retrospective design. Another limitation of this study is that it was a single-center study, and all patients included in this study were of Han ethnicity.

Therefore, we would advise that all patients with LN undergo testing for ANCAs and other autoantibodies that mediate glomerular pathology, including anti-cardiolipin and anti-glomerular basement membrane antibodies. We also need additional prospective studies to determine whether long-term outcomes are different between the ANCA-positive and ANCA-negative groups, as this will guide the appropriate screening, monitoring, and treatment of these patients.
